# A Case of Reproduction of a Portion of the Lower Jaw, with the Teeth

**Published:** 1853-04

**Authors:** J. M. Todd

**Affiliations:** Monongahela City, Pa.


*A case of Reproduction of a portion of the Lower Jaw, with the Teeth.* By Dr. J. M. TODD, of Monongahela city, Pa.

DR. TAYLOR, DEAR SIR :—I drop you a line about a very extraordinary case of second development of the lower jaw. The history of the case is this. A young lad, aged about twelve years, had his first molar, on the left side below, extracted, a slight fracture of the jaw occurred, which resulted in caries. A considerable discharge took place for about eighteen months. Drs. Biddell and Keys, failing to cure by every means, usually employed in such cases, resolved upon its removal. They accordingly proceeded, and on making a free incission from about the region of the ear to within a short distance of the base of the lower jaw, the carious portion, which comprised about one half the bone, *dropped out.* All diseased portions of bone being removed, the wound was closed, and the parts healed kindly. In the course of a few days, quite a deposit, of what appeared to be bone, took the place of the removed portion of bone, this grew and hardened, finally ossified, the articulation seeming *complete.* The *use* of the jaw was restored, with but a very little deformity, which consisted of a slight enlargement of the face on the afflicted side, a *little too much new bone,* and a slight twist of the mouth. But the "wonder of wonders" is yet to be told. The boy is, *now* getting a *new set of teeth* on that side also. The first and second molars being *already through, with a fair prospect* of the rest soon "*following suit.*"

You will understand that the *entire half* of the bone was removed. I have scribbled this off in a great hurry, if you desire a more particular history of the case, I will give it to you at any time. Though you here have the skeleton. The case is still doing well. The use of the jaw is entirely restored. *I dont know* that there is a similar case on record. At any rate, it gives us something to think and talk about, as it rather conflicts with our previous notions of the dental development. To say the least of it, it is *a very strange, unaccountable, lussus naturæ.**

*We hope that Dr. Todd will watch the further progress of this interesting case, and let us know how many teeth are reproduced. If they have a perfect alveolus, and if the teeth are of the class which should occupy that portion of the jaw, &c. &c.

